# Human Physiological Responses to a Single Deep Helium-Oxygen Diving

**DOI:** 10.3389/fphys.2021.735986

**Published:** 2021-09-28

**Authors:** Xiao-Chen Bao, Quan Shen, Yi-Qun Fang, Jian-guo Wu

**Affiliations:** ^1^Department of Diving and Hyperbaric Medicine, Naval Medical Center, Shanghai, China; ^2^Department of Hyperbaric Medicine, Naval Hospital of Eastern Theater, Zhejiang, China

**Keywords:** diving, heliox, endothelial (dys)function, oxidative stress, physiological stress

## Abstract

**Objective:** The objective of this study was to explore whether a single deep helium-oxygen (heliox) dive affects physiological function.

**Methods:** A total of 40 male divers performed an open-water heliox dive to 80 m of seawater (msw). The total diving time was 280 min, and the breathing helium-oxygen time was 20 min. Before and after the dive, blood and saliva samples were collected, and blood cell counts, cardiac damage, oxidative stress, vascular endothelial activation, and hormonal biomarkers were assayed.

**Results:** An 80 msw heliox dive induced a significant increase in the percentage of granulocytes (GR %), whereas the percentage of lymphocytes (LYM %), percentage of intermediate cells (MID %), red blood cell number (RBC), hematocrit (hCT), and platelets (PLT) decreased. During the dive, concentrations of creatine kinase (CK), a myocardial-specific isoenzyme of creatine kinase (CK-MB) in serum and amylase alpha 1 (AMY1), and testosterone levels in saliva increased, in contrast, IgA levels in saliva decreased. Diving caused a significant increase in serum glutathione (GSH) levels and reduced vascular cell adhesion molecule-1 (VCAM-1) levels but had no effect on malondialdehyde (MDA) and endothelin-1 (ET-1) levels.

**Conclusion:** A single 80 msw heliox dive activates the endothelium, causes skeletal-muscle damage, and induces oxidative stress and physiological stress responses, as reflected in changes in biomarker concentrations.

## Introduction

Adverse environmental conditions, such as elevated ambient pressure, immersion, cold, hyperoxia, and changes in breathing-gas characteristics, cause stress during diving (Pendergast et al., 2015). To maintain homeostasis, the body activates molecular mechanisms that lead to functional adaptations in, for instance, the heart, lung (Skogstad et al., 2002), muscles (Žarak et al., 2021), blood vessels (Guerrero et al., 2020), and activation of the immune system (Sureda et al., 2014).

Several asymptomatic changes occur following a dive. Single recreational scuba diving (30 m depth) (Žarak et al., 2020) and technical diving (55–80 m depth) (Marinovic et al., 2010) trigger the changes in biomarkers of cardiac damage and vascular endothelial activation. Changes in lung diffusion properties, reduction in spirometric parameters, and accumulation of extravascular lung water were detected following dives to depths of 50–80 m (Marinovic et al., 2010). Both cold and depth contributed to the adverse effects of a single compressed-air dive (50 m depth) on pulmonary function (Tetzlaff et al., 2001). Reactive oxygen species (ROS) are involved in the vascular impairment linked to endothelial dysfunction during scuba dives (55–80 m depth) (Obad et al., 2010). Levels of physiological stress markers, such as the stress hormones, prolactin (Anegg et al., 2002), and cortisol, increased with immersion depth (1–30 m depths) (Zarezadeh and Azarbayjani, 2014).

Helium is less soluble and more diffusive than nitrogen. Helium-oxygen (heliox) mixtures are used as a breathing medium during deep dives to avoid the narcotic effects of nitrogen under pressure. Endothelial dysfunction in pulmonary circulation was found in rats following a single simulated heliox dive (Berenji Ardestani et al., 2019). There are few studies on the effects of heliox diving on human physiological functions. Acute endothelial dysfunction in the large conduit arteries occurred after successive deep trimix dives (55–80 m) (Obad et al., 2010). The depressed systolic function of the left side of the heart was found in divers after trimix diving to a depth of 55 m (Marinovic et al., 2009). Marinovic reported that trimix dives (55–80 m) promoted accumulation of extravascular lung water, diminished left ventricular contractility, and increased release of N terminal pro B type natriuretic peptide (NT-proBNP; Marinovic et al., 2010). However, information on the effect of deep heliox diving on physiological indicators is scarce.

To investigate the effects of a single deep heliox dive on the body, we evaluated the changes in peripheral blood cells, plasma concentrations of myocardial enzymes, oxidative stress, vascular endothelial activation, and physiological stress biomarkers before and after an 80 m heliox dive.

## Materials and Methods

### Subjects

A total of 40 healthy male navy divers, aged 18–32 years with diving experience of 2–12 years, were recruited. All experimental procedures were performed in accordance with the Declaration of Helsinki and were approved by the Ethical Committee of the Naval Hospital of Eastern Theater (protocol code 202008). All subjects provided written informed consent. Health status and previous diving experience were self-reported. All divers met “The Medical Examination Standards for Professional Divers” (China National Standard, GB 20827-2007, 2007.08.01). Failure to meet the physical examination standards of divers, or a history of cold or Eustachian tube dysfunction in the past week were the exclusion criteria.

### Diving Decompression Scheme

To reduce water-decompression and total-decompression times, surface decompression with oxygen (SURDO_2_) was used. SURDO_2_ is a technique for fulfilling all or part of the decompression obligations of divers in a recompression chamber instead of in the water. A shorter time in the water prevents a dangerous reduction in body temperature. Inside the hyperbaric oxygen chamber, the divers can be maintained at constant pressure, unaffected by sea-surface conditions.

**FIGURE 1:**
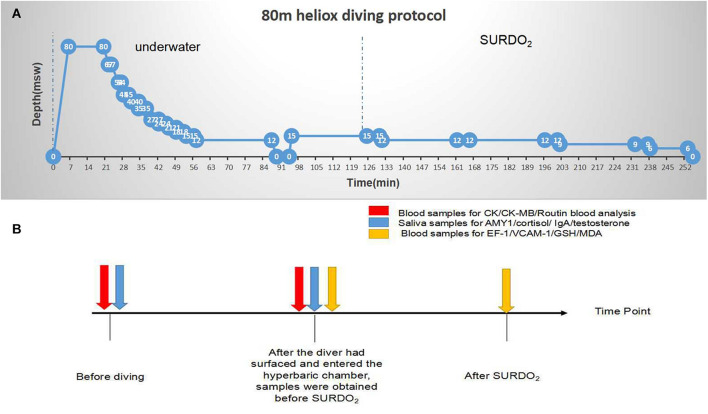
Decompression procedure for 80 m heliox diving and sampling collection time points. **(A)** Decompression procedure for 80 m heliox diving. After blood and saliva collection, divers immersed to 80 msw at a rate of 12 msw/s, breathing air to heliox (He:O_2_, 82:18) with KMB 28B surface-supplied diving equipment. The bottom time was 15 min, and divers returned to the first stop station (67 msw) at an ascending velocity of 6 msw/min. After converting their breathing gas from heliox to air at 45 msw, divers ascended to 12 msw following the decompression table. After inhaling oxygen for 30 min at the 12 msw stop station, divers returned to the surface in 6 min and completed surface decompression with oxygen in a hyperbaric oxygen chamber. At the end of the experiment, blood and saliva samples were collected, and blood cell numbers and biomarkers were assayed. **(B)** Sampling collection time points.

As shown in Figure 1A, each diver, wearing a wet suit and breathing air to heliox (He:O_2_, 82:18) with an open-circuit breathing apparatus (KMB 28B diving mask), gradually descended to 80 m of the seawater (msw) at 12 m/min. The surface water temperature was 26°C, the bottom water temperature was 16°C, the wave height was 1.0–1.5 m, and the water flow was <0.5 m/s. The time at the bottom was 15 min, and the diver returned to the first stop station (67 msw) at 6 m/min. After converting the breathing gas from heliox to air at 45 msw, the diver ascended to 12 msw following the decompression table (Figure 1). After inhaling oxygen for 30 min at the 12 msw stop station, the diver returned to the surface within 6 min and recompressed to 15 msw in a hyperbaric oxygen chamber, breathing oxygen. The interval between surface and recompression was controlled at 5 min. The SURDO_2_ procedure is shown in Figure 1A. Some divers cruised at 83 msw underwater. These divers added a cycle of oxygen uptake at a depth of 12 msw in the hyperbaric oxygen chamber.

### Saliva Sampling

As shown in Figure 1B, saliva and blood samples were collected during the dive. Unstimulated whole saliva samples (*n* = 40) were obtained before diving and after surfacing and entering the recompression cabin. The mouth was rinsed with clean water 30 min before saliva collection, the tongue was pressed against the palate to enrich the saliva, and the saliva was spat into a sterile polypropylene transfer pipette (2 ml). No buffer was added to the collection tube. After sample collection (before and after diving, separately), the samples were immediately transported on ice to the laboratory and centrifuged at 4,000 rpm for 10 min at 4°C. The supernatant was aliquoted into storage vials and stored in liquid nitrogen until analysis.

### Blood Sampling

For blood cell counts and myocardial enzyme analysis, samples were obtained before diving (baseline) and after surfacing and entering the recompression cabin (*n* = 40, Figure 1B). Venous blood specimens (1 ml) were obtained from the superficial vein of the elbow into vacutainers containing ethylene diamine tetraacetic acid (EDTA) as an anticoagulant (Becton, Dickinson and Company, Franklin Lakes, NJ, United States) and analyzed using a blood counter (Abbott, IL, United States). One milliliter of the sample was used to assay creatine kinase (CK) and myocardial-specific isoenzyme of creatine kinase (CK-MB) activities.

After the diver had surfaced and entered the hyperbaric chamber, 2 ml of blood samples were obtained before SURDO_2_. A further 2 ml of blood sample was obtained on completion of SURDO_2_. Blood samples were drawn into tubes without anticoagulants and placed at 4°C for 2 h. Then, the samples were centrifuged at 1,000 *g* at 4°C for 20 min. The supernatant was stored at −80°C until delivery to the laboratory for measurement of glutathione (GSH) activity and malondialdehyde (MDA), endothelin-1 (ET-1), and vascular cell adhesion molecule-1 (VCAM-1) levels.

### Hormonal Stress Markers Test

Serum levels of ET-1 and VCAM-1 (Cusabio Biotech Co., Ltd., Wuhan, China) and saliva levels of amylase alpha 1 (AMY1), cortisol, IgA, and testosterone were evaluated by enzyme-linked immunosorbent assay (ELISA; Elabscience Biotechnology Co., Ltd., Wuhan, China). Levels of MDA and GSH were determined by chemical colorimetry using commercial assay kits (Nanjing Jiancheng Bioengineering Institute, Nanjing, China). All assays were performed in accordance with the respective instructions of the manufacturer.

### Statistical Analysis

Statistical analyses were performed using SPSS version 11.5 software (SPSS, Inc., Chicago, IL, United States). Results are means ± SD. A paired-sample *t*-test (two-tailed) and unpaired *t*-test with Welch’s correction were performed based on the normality of the distribution as checked by using the Shapiro–Wilk test. A *P*-value of <0.05 was considered indicative of statistical significance.

## Results

### Changes in Blood Cell Counts

**FIGURE 2:**
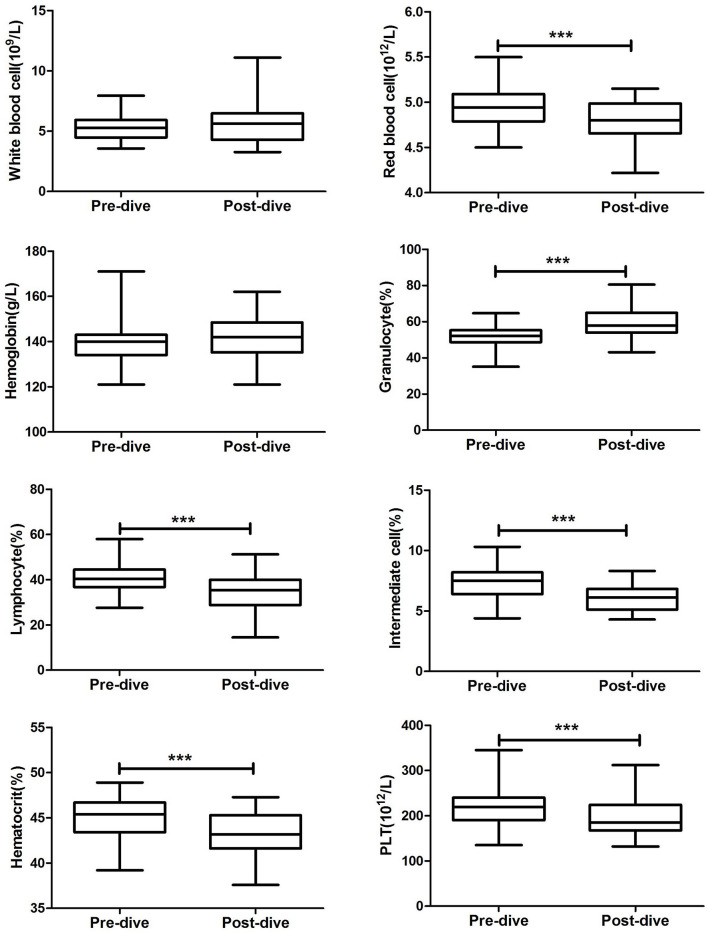
Changes in peripheral blood cells before and after 80 m heliox diving. ***P < 0.0001 compared with predive. Data are means ± SD (*n* = 40). The paired-sample *t*-test (two-tailed) was performed based on the normality of the distribution as checked by using the Shapiro–Wilk test.

After the dive, all divers felt normal, and none complained of physical discomfort. An 80 msw heliox dive caused significant decreases in numbers of red blood cells (RBCs, 4.79 ± 0.23 vs. 4.97 ± 0.22 × 10^12^/L, *P* < 0.0001; Figure 2), platelets (PLT, 196.1 ± 41.84 vs. 223.0 ± 42.74 × 10^9^/L, *P* < 0.0001), percentage of lymphocytes (LYM %, 34.28 ± 8.67 vs. 41.58 ± 6.65%, *P* < 0.0001), percentage of intermediate cells (MID %, 6.20 ± 1.04 vs. 7.29 ± 1.30%, *P* < 0.0001), and hematocrit (hCT, 43.22 ± 2.35 vs. 45.21 ± 2.04%, *P* < 0.0001). The percentage of granulocytes increased markedly during the dive (GR %, 59.52 ± 8.84 vs. 51.13 ± 6.63%, *P* < 0.0001). Diving caused slight but non-significant increases in hemoglobin (HGB, 141.6 ± 8.98 vs. 140.1 ± 9.26 g/L, *P* = 0.30) and white blood cell count (WBC, 5.63 ± 1.61 vs. 5.20 ± 1.01 × 10^9^/L, *P* = 0.07).

### Changes of Myocardial Enzyme Spectrum

**FIGURE 3:**
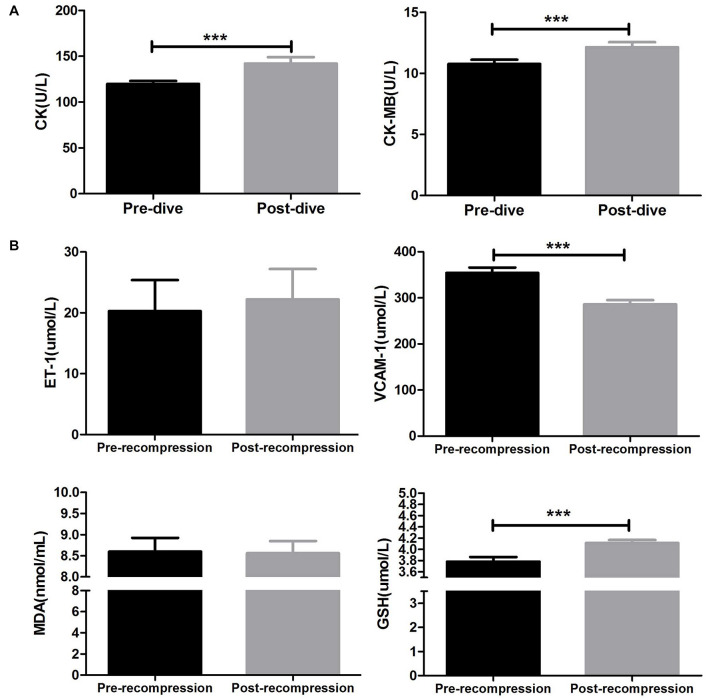
Changes in myocardial enzymes and serum indices before and after 80 m heliox diving. **(A)** Creatine kinase (CK) and myocardial-specific isoenzyme of creatine kinase (CK-MB) decreased significantly after ascent to the surface. ***P < 0.0001 compared with the predive group. The CK and CK-MB data met the homogeneity of variance and were analyzed using a paired *t*-test. **(B)** Changes in endothelin-1 (ET-1), vascular cell adhesion molecule-1 (VCAM-1), serum glutathione (GSH), and malondialdehyde (MDA) concentrations between before and after recompression treatment in a hyperbaric oxygen chamber. ***P < 0.0001 compared with pretreatment. Data are means ± SD (*n* = 40). The ET-1, VCAM-1, GSH, and MDA data did not meet the homogeneity of variance and were analyzed using an unpaired *t*-test with Welch’s correction.

As indicated in Figure 3A, compared with baseline, the mean CK level significantly increased after surfacing (142.1 ± 44.02 vs. 120 ± 19.94 U/L; *P* = 0.0003). Also, 80 m heliox diving caused a significant increase in the mean CK-MB level (12.14 ± 2.65 vs. 10.77 ± 2.20 U/L, *P* < 0.0001).

### Changes of Serum Indexes

As shown in Figure 3B, GSH activity in plasma was significantly higher after SURDO_2_ (4.11 ± 0.47 vs. 3.78 ± 0.72 μmol/L, *P* = 0.007), but there was no significant change in MDA activity between before and after SURDO_2_ (8.56 ± 2.54 vs. 8.60 ± 2.91 nmol/L; *P* = 0.76). The mean expression level of VCAM-1 decreased significantly after SURDO_2_ (286.0 ± 81.54 vs. 354.1 ± 103.6 μmol/L, *P* < 0.0001). However, SURDO_2_ had no significant effect on the mean ET-1 level (23.32 ± 48.54 vs. 22.22 ± 44.14 μmol/L; *P* = 0.78, Figure 3B).

### Changes of Hormonal Stress Markers

**FIGURE 4:**
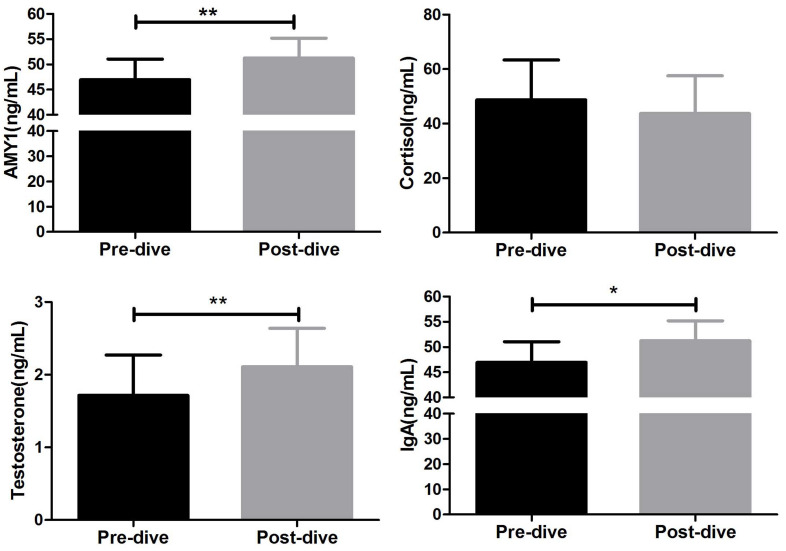
Changes in hormonal indicators between before and after 80 m heliox diving. *P < 0.05, **P < 0.01 compared with the predive group. Data are expressed as means ± SD (*n* = 40). The AMY1 data met the homogeneity of variance and were analyzed using a paired *t*-test, whereas the cortisol, testosterone, and IgA data did not and were subjected to an unpaired *t*-test with Welch’s correction.

The changes in AMY1, cortisol, IgA, and testosterone levels in saliva between before and after diving are shown in Figure 4. Diving markedly increased the mean saliva AMY1 level (51.21 ± 25.28 vs. 46.94 ± 26.04 ng/ml; *P* = 0.007). The changes in mean testosterone (2.11 ± 3.36 vs. 1.72 ± 3.52 ng/ml; *P* = 0.008) and IgA (646.2 ± 397.5 vs. 756.0 ± 506.0 ng/ml; *P* = 0.024) levels showed the same trend, but the mean cortisol level did not change significantly between before and after diving (43.73 ± 83.10 vs. 48.72 ± 87.96 ng/ml; *P* = 0.805, Figure 4).

## Discussion

### Inflammatory Response and Platelet Activation

Deep heliox diving resulted in an increase and decrease, respectively, in granulocyte and lymphocyte numbers immediately after surfacing. Deep heliox diving involves exposure to several physiological and psychological stress factors, e.g., elevated ambient pressure, immersion, cold, and breathing of hyperbaric oxygen (HBO) and inert gasses. Physiological stress may contribute to neutrophil mobilization by increasing the release of stress hormones such as cortisol (Anegg et al., 2002). The unbalanced endocrine/immunological response induced during diving alters the proportions of circulating blood cells. Furthermore, microbubbles produced during the decompression process induce endothelial damage and affect leukocyte mobilization (Glavas et al., 2008).

Granulocyte increase is often accompanied by a mild increase in monocytes in the inflammatory response (Natale et al., 2003). However, in this study, the mean number of intermediate cells decreased immediately after surfacing, which was inconsistent with Perovic’s study (Perovic et al., 2017). They found that neutrophils increased and monocytes decreased immediately after 30 m-depth compressed-air diving. Since the number of intermediate cells in humans is small, the reduction in the percentage of intermediate cells may be a transient response to external stimuli. This may be caused by *trans-*endothelial migration due to altered vascular/endothelial function after diving (Obad et al., 2010).

Diving is a hyperoxic physical activity under high pressure, which can induce oxidative stress by increasing free-radical production. Since RBC membranes are highly susceptible to peroxidative damage, they are sensitive to oxidative stress (Santos-Silva et al., 2001). In this study, deep heliox diving caused a significant decrease in RBC and hCT but had no significant effect on HGB levels. These changes can be explained by oxidative-damage-induced fragility of the RBC membrane. The number of RBCs subsequently decreases, causing a reduction in hematocrit.

Changes in PLT counts after hyperbaric exposure and decompression have been reported in animal studies (Pontier et al., 2009; Lambrechts et al., 2018) and in divers without clinical symptoms (Olszański et al., 2001). In animal models of decompression sickness, circulating bubbles damage the vascular wall and activate endothelial cells. Exposed collagen and the subendothelial basal lamina activate the coagulation system, which forms microthrombi and reduces the platelet count (Eckmann and Armstead, 2006). Platelets adhere to the bubble surface and are activated (Olszański et al., 1997). The postdive decrease in platelet count is related to decompression-induced bubble formation (Pontier and Lambrechts, 2014). However, in the above-mentioned studies, the objective, diving depth, and decompression methods differed. We conclude that decompression bubbles caused the PLT reduction. In this study, divers did not show symptoms of decompression sickness, but asymptomatic microbubbles may have been present. Therefore, the reduction in PLT may have been caused by asymptomatic air bubbles during decompression; however, this needs to be confirmed. Platelet functions, such as aggregation and adhesion, need to be assayed in a future study.

### Muscle Injury

Creatine kinase is found mainly in the skeletal muscle, cardiac muscle, and brain. CK and CK-MB elevations are markers of arterial gas embolism (Smith and Neuman, 1994). However, in this study, although CK and CK-MB increased significantly after diving, the values remained within the normal range. This implies that the CK and MB isoenzyme have a non-myocardial source. CK activity increased in a bubble-free scuba dive (Bilopavlovic et al., 2013), so in this study, we speculate that the elevations in CK and CK-MB were mainly from skeletal muscle.

### Physiological Stress Response

Scuba dives induce increased levels of stress hormones (Anegg et al., 2002) and cortisol according to immersion depth (Zarezadeh and Azarbayjani, 2014). Such dives are associated with transient endothelial dysfunction, which is likely mediated by hyperbaric O_2_, and have been linked to reduced cognitive performance (Pourhashemi et al., 2016) and stress cardiomyopathy (Beneton et al., 2017).

Cortisol is used to assess the stress response. An increase in cortisol levels is associated with anxiety, depression, and intensive physical exercise. Reports of changes in cortisol concentrations following hyperbaric exposure are contradictory. Some found that scuba diving increased cortisol levels (Pourhashemi et al., 2016). However, in other studies, cortisol levels did not change or decrease after scuba dives (Marlinge et al., 2019). In this study, saliva cortisol levels had a non-significantly decreasing trend after surfacing. This is at odds with reported increased saliva cortisol and decreased cognitive performance (Pourhashemi et al., 2016), possibly due to differences in diving methods in the two studies. In addition, the hyperbaric O_2_ inhaled during SURDO_2_ might have counterbalanced the effect of the cold immersion response by reducing physiological stress levels.

Testosterone is an androgen that influences athletic ability and psychological activity. The data on changes in testosterone concentrations after diving are conflicting. Jóźków et al. (2012) found that breath-hold diving did not affect the secretion of gonadotropins or androgen concentrations. Hyperbaric- but not hyperoxic-induced testosterone reductions were observed in scuba divers by Verratti et al. (2019). However, hyperbaric oxygen therapy can increase blood testosterone concentrations (Passavanti et al., 2010). The diving depth and differences in diving methods may explain the inconsistency of changes in testosterone levels. In this study, testosterone levels increased markedly after diving, likely because heliox divers are exposed to higher absolute levels of oxygen. In breath-hold diving, hypoxia and hypercapnia are the main determinants of the physiological response.

In mucosal immunity in the upper respiratory tract, IgA plays an important role. It inhibits the adhesion of microorganisms to the upper respiratory tract epithelium, slowing their proliferation. Volozhin et al. (2001) found that saturation diving decreased IgA and S-IgA levels. Short-term operational heliox exposure did not affect IgA levels (Olszański et al., 2002). During saturation diving, divers spend a long time underwater, which can suppress immune function. In Olszański’s study, the diving time was too short to affect IgA levels. In this study, IgA levels increased markedly after diving, which is likely in response to diving stress.

Salivary AMY1 catalyzes the hydrolysis of starch to maltose. The secretion of salivary amylase is regulated by the sympathetic nervous system. When the system is excited, the secretion of salivary amylase increases, and the reaction speed is faster than that of noradrenaline, cortisol, and other hormones (Jones et al., 2020). In this study, salivary AMY1 levels increased markedly after diving. Similarly, Gilman et al. (1979) found that secretion of alpha-amylase by the human parotid gland increased during 8 days of hyperbaric exposure. Enhanced autonomic nervous system (ANS) activity has been attributed to increased physiological stress.

### Oxidative Stress

In diving, excess oxidative stress is caused by physical and chemical stress factors in the hyperbaric environment (Obad et al., 2007). Due to the increased exhalation resistance and low temperatures underwater, scuba diving is a high-strength activity. Intensified physical activity, hyperoxia, and exposure to low temperatures lead to the production of free radicals (Ferrer et al., 2007). In this study, GSH levels increased significantly during diving. GSH, an abundant tripeptidyl molecule, protects cells against oxidative-stress-induced cellular damage and detoxifies xenobiotics and drugs. As a marker of lipid peroxidation, MDA is used as an indicator of oxidative damage. Dujic found that MDA values increased after successive deep trimix dives to depths of 55–80 m (Obad et al., 2010). However, in this study, MDA values did not change between before and after SURDO_2_. There are two possible reasons. First, because GSH and MDA were measured before and after oxygen inhalation in the hyperbaric chamber (SURDO_2_), we think that the increase in GSH was due to the response of the body to hyperoxia exposure. The lack of change in MDA activity indicates that the oxidative stress induced by 80 m heliox diving (underwater exposure) did not induce cell damage. Second, in Dujic’s study, the divers breathed a gas mixture containing a high concentration of oxygen during the decompression phase (>30%); this concentration was much higher than in this study (18%).

### Endothelium Damage

In human and animal models, diving can cause endothelial dysfunction. Bubbles formed during decompression interact with, and damage, the endothelium. Oxidative stress induced by hyperoxia also leads to dysfunction of the endothelium (Berenji Ardestani et al., 2019). In this study, we assayed VCAM-1 and ET-1 levels to explore changes in endothelial function after deep helium-oxygen diving. ET-1 is a vasoactive substance secreted by endothelial cells that is a marker of endothelial injury; ET-1 levels did not change significantly after diving. Since excessive production of ET-1 is an indicator of altered endothelial function (Iglarz and Clozel, 2007), the lack of change in ET-1 levels implies that the endothelial damage was not sufficiently severe as to be reflected by detectable changes in blood markers. Since the plasma half-life of ET-1 is 4–7 min, another possible explanation for the lack of change in ET-1 levels is that it was missed.

Vascular cell adhesion molecule-1 is expressed exclusively on activated endothelium following vascular insult, and therefore, is a marker of proinflammatory endothelium. In one study, VCAM-1 levels increased post-decompression in DCI rats (Zhang et al., 2016). Vince et al. (2009) found that a simulated dive of 78 min bottom time at 2.8 absolute atmospheric pressure (ATA) significantly increased VCAM^+^ MP, and oxidative stress was correlated with VCAM^+^ MP. Compared with before SURDO_2_, VCAM-1 levels were significantly reduced after SURDO_2_, implying that heliox diving (underwater process) caused endothelial damage, which improved after inhalation of oxygen during SURDO_2_.

### Comparing With Other Extremes of Environment

In Table 1, we compare the changes of physiological indicators in different extremes of the environment, such as high altitude and microgravity. Hypoxia is the main feature in high-altitude environments. In space, the special conditions of hypogravity and exposure to cosmic radiation have an impact on the human body and human organ functions. Harmful factors in different environments cause corresponding changes in the physiological indicators of the body.

**TABLE 1:** The impact of different extreme environments on physiological indicators.

	Deep diving	High altitude	Space flight
Blood cell counts	Decrease RBC, PLT, LYM, MID, and hCT	Changes in hematocrit, red blood count (Mairbäurl, 1994)	Plasma volume decrease, red blood cell mass decrease (Alfrey et al., 1996)
Skeletal-muscle	CK, CK-MB increased	High altitude mitochondrial volume density is reduced (Stienen, 2020)	Muscle and bone atrophy
Oxidative stress	GSH increased, MDA had no change	Reactive oxygen species (ROS) production is accelerated with mountainous elevation (Gaur et al., 2021)	Generation of ROS, leading to a dysregulation in the oxidants-antioxidants balance (Pavlakou et al., 2018)
Hormonal stress	AMY1, testosterone, IgA increased	Testosterone levels increase with mountainous elevation (Dittmar, 2014)	Plasma and urinary cortisol increase (Stowe et al., 2011), Testosterone was not changed during long-duration space flight but were decreased on landing day (Smith et al., 2012)
Endothelial damage	VCAM-1 increased after heliox diving (underwater process), which improved after inhalation of oxygen during SURDO_2_	Non-uniform regional hypoxic arteriolar vasoconstriction induce endothelial damage (Swenson and Bärtsch, 2012)	The upregulation of VCAM-1 may contribute to impaired endothelium-dependent relaxation in simulated microgravity rat vasculature (Zhang et al., 2008)

## Conclusion

Eighty-meter open-water heliox diving induced endothelial and skeletal-muscle damage, oxidative stress, and physiological stress responses, as reflected in changes in biomarker concentrations.

## Limitations

Since divers must be transferred to a hyperbaric oxygen chamber quickly to complete surface decompression after ascending, we did not assess decompression-induced microbubbles after diving. Future studies would benefit from the assessment of bubble formation.

## Data Availability Statement

The original contributions presented in the study are included in the article/Supplementary Material, further inquiries can be directed to the corresponding author.

## Ethics Statement

The studies involving human participants were reviewed and approved by the Ethical Committee of the Naval Hospital of Eastern Theater. The patients/participants provided their written informed consent to participate in this study.

## Author Contributions

X-CB and Y-QF conceived and designed the study and drafted the manuscript. X-CB, QS, and J-GW performed the experiments. X-CB and QS analyzed the data. Y-QF approved the final version of the manuscript. All authors contributed to the article and approved the submitted version.

## Conflict of Interest

The authors declare that the research was conducted in the absence of any commercial or financial relationships that could be construed as a potential conflict of interest.

## Publisher’s Note

All claims expressed in this article are solely those of the authors and do not necessarily represent those of their affiliated organizations, or those of the publisher, the editors and the reviewers. Any product that may be evaluated in this article, or claim that may be made by its manufacturer, is not guaranteed or endorsed by the publisher.

## References

[B1] Alfrey C. P. Udden M. M. Leach-Huntoon C. Driscoll T. Pickett M. H. (1996). Control of red blood cell mass in spaceflight. *J. Appl. Physiol. (Bethesda, Md : 1985)* 81 98–104. 10.1152/jappl.1996.81.1.98 8828651

[B2] Anegg U. Dietmaier G. Maier A. Tomaselli F. Gabor S. Kallus K. W. (2002). Stress-induced hormonal and mood responses in scuba divers: a field study. *Life Sci.* 70 2721–2734. 12269378 10.1016/s0024-3205(02)01537-0

[B3] Beneton F. Michoud G. Coulange M. Laine N. Ramdani C. Borgnetta M. (2017). Recreational diving practice for stress management: an exploratory trial. *Front. Psychol.* 8:2193. 10.3389/fpsyg.2017.02193 29326628 PMC5741699

[B4] Berenji Ardestani S. Matchkov V. V. Eftedal I. Pedersen M. A. (2019). Single simulated heliox dive modifies endothelial function in the vascular wall of apoe knockout male rats more than females. *Front. Physiol.* 10:1342. 10.3389/fphys.2019.01342 31695628 PMC6817487

[B5] Bilopavlovic N. Marinovic J. Ljubkovic M. Obad A. Zanchi J. Pollock N. W. (2013). Effect of repetitive SCUBA diving on humoral markers of endothelial and central nervous system integrity. *Eur. J. Appl. Physiol.* 113 1737–1743. 10.1007/s00421-013-2600-4 23400567

[B6] Dittmar M. (2014). Human biological research since 2006 at the Christian-Albrechts-University in Kiel–aging, chronobiology, and high altitude adaptation. *Anthropol. Anz.* 71 143–153. 10.1127/0003-5548/2014/0392 24818445

[B7] Eckmann D. M. Armstead S. C. (2006). Influence of endothelial glycocalyx degradation and surfactants on air embolism adhesion. *Anesthesiology* 105 1220–1227. 10.1097/00000542-200612000-00022 17122585

[B8] Ferrer M. D. Sureda A. Batle J. M. Tauler P. Tur J. A. Pons A. (2007). Scuba diving enhances endogenous antioxidant defenses in lymphocytes and neutrophils. *Free Radic. Res.* 41 274–281. 10.1080/10715760601080371 17364955

[B9] Gaur P. Prasad S. Kumar B. Sharma S. K. Vats P. (2021). High-altitude hypoxia induced reactive oxygen species generation, signaling, and mitigation approaches. *Int. J. Biometeorol.* 65 601–615. 10.1007/s00484-020-02037-1 33156424

[B10] Gilman S. C. Fischer G. J. Biersner R. J. Thornton R. D. Miller D. A. (1979). Human parotid alpha-amylase secretion as a function of chronic hyperbaric exposure. *Undersea Biomed. Res.* 6 303–307. 316598

[B11] Glavas D. Markotic A. Valic Z. Kovacic N. Palada I. Martinic R. (2008). Expression of endothelial selectin ligands on human leukocytes following dive. *Exp. Biol. Med. (Maywood, NJ)* 233 1181–1188. 10.3181/0801-rm-28 18535169

[B12] Guerrero F. Lambrechts K. Wang Q. Mazur A. Théron M. Marroni A. (2020). Endothelial function may be enhanced in the cutaneous microcirculation after a single air dive. *Diving Hyperb. Med.* 50 214–219. 10.28920/dhm50.3.214-219 32957122 PMC7819729

[B13] Iglarz M. Clozel M. (2007). Mechanisms of ET-1-induced endothelial dysfunction. *J. Cardiovasc. Pharmacol.* 50 621–628. 18091577 10.1097/FJC.0b013e31813c6cc3

[B14] Jones E. J. Rohleder N. Schreier H. M. C. (2020). Neuroendocrine coordination and youth behavior problems: a review of studies assessing sympathetic nervous system and hypothalamic-pituitary adrenal axis activity using salivary alpha amylase and salivary cortisol. *Horm. Behav.* 122:104750. 10.1016/j.yhbeh.2020.104750 32302595

[B15] Jóźków P. Mędraś M. Chmura J. Kawczyński A. Morawiec B. (2012). Effect of breath-hold diving (freediving) on serum androgen levels – a preliminary report. *Endokrynol. Polska* 63 381–387. 23115072

[B16] Lambrechts K. de Maistre S. Abraini J. H. Blatteau J. E. Risso J. J. Vallée N. (2018). Tirofiban, a glycoprotein IIb/IIIa antagonist, has a protective effect on decompression sickness in rats: is the crosstalk between platelet and leukocytes essential? *Front. Physiol.* 9:906. 10.3389/fphys.2018.00906 30050468 PMC6050390

[B17] Mairbäurl H. (1994). Red blood cell function in hypoxia at altitude and exercise. *Int. J. Sports Med.* 15 51–63. 10.1055/s-2007-1021020 8157369

[B18] Marinovic J. Ljubkovic M. Obad A. Bakovic D. Breskovic T. Dujic Z. (2009). Effects of successive air and trimix dives on human cardiovascular function. *Med. Sci. Sports Exerc.* 41 2207–2212. 10.1249/mss.0b013e3181aa04cc 19915497

[B19] Marinovic J. Ljubkovic M. Obad A. Breskovic T. Salamunic I. Denoble P. J. (2010). Assessment of extravascular lung water and cardiac function in trimix SCUBA diving. *Med. Sci. Sports Exerc.* 42 1054–1061. 19997032 10.1249/MSS.0b013e3181c5b8a8

[B20] Marlinge M. Coulange M. Fitzpatrick R. C. Delacroix R. Gabarre A. Lainé N. (2019). Physiological stress markers during breath-hold diving and SCUBA diving. *Physiol. Rep.* 7:e14033. 10.14814/phy2.14033 30912280 PMC6434169

[B21] Natale V. M. Brenner I. K. Moldoveanu A. I. Vasiliou P. Shek P. Shephard R. J. (2003). Effects of three different types of exercise on blood leukocyte count during and following exercise. *Sao Paulo med. J. Rev. Paulista Med.* 121 9–14. 10.1590/S1516-31802003000100003 PMC11108609 12751337

[B22] Obad A. Marinovic J. Ljubkovic M. Breskovic T. Modun D. Boban M. (2010). Successive deep dives impair endothelial function and enhance oxidative stress in man. *Clin. Physiol. Funct. Imaging* 30 432–438. 10.1111/j.1475-097x.2010.00962.x 20718805

[B23] Obad A. Palada I. Valic Z. Ivancev V. Baković D. Wisløff U. (2007). The effects of acute oral antioxidants on diving-induced alterations in human cardiovascular function. *J. Physiol.* 578(Pt 3) 859–870. 10.1113/jphysiol.2006.122218 17110413 PMC2151345

[B24] Olszański R. Konarski M. Kierznikowicz B. (2002). Changes of selected morphotic parameters and blood plasma proteins in blood of divers after a single short-time operational heliox exposure. *Int. Marit. Health* 53 111–121. 12608594

[B25] Olszański R. Radziwon P. Baj Z. Kaczmarek P. Giedrojć J. Galar M. (2001). Changes in the extrinsic and intrinsic coagulation pathways in humans after decompression following saturation diving. *Blood Coagul. Fibrinolysis* 12 269–274. 10.1097/00001721-200106000-00007 11460010

[B26] Olszański R. Sićko Z. Baj Z. Czestochowska E. Konarski M. Kot J. (1997). Effect of saturated air and nitrox diving on selected parameters of haemostasis. *Bull. Inst. Marit. Trop. Med. Gdynia* 48 75–82. 9591152

[B27] Passavanti G. Tanasi P. Brauzzi M. Pagni M. R. Aloisi A. M. (2010). Can hyperbaric oxygenation therapy (HOT) modify the blood testosterone concentration? *Urologia* 77 52–56. 10.1177/039156031007700109 20890859

[B28] Pavlakou P. Dounousi E. Roumeliotis S. Eleftheriadis T. Liakopoulos V. (2018). Oxidative Stress and the Kidney in the Space Environment. *Int. J. Mol. Sci.* 19:3176. 10.3390/ijms19103176 30326648 PMC6214023

[B29] Pendergast D. R. Moon R. E. Krasney J. J. Held H. E. Zamparo P. (2015). Human physiology in an aquatic environment. *Compr. Physiol.* 5 1705–1750. 10.1002/cphy.c140018 26426465

[B30] Perovic A. Nikolac N. Braticevic M. N. Milcic A. Sobocanec S. Balog T. (2017). Does recreational scuba diving have clinically significant effect on routine haematological parameters? *Biochem. Med.* 27 325–331. 10.11613/BM.2017.035 28694723 PMC5493166

[B31] Pontier J. M. Lambrechts K. (2014). Effect of oxygen-breathing during a decompression-stop on bubble-induced platelet activation after an open-sea air dive: oxygen-stop decompression. *Eur. J. Appl. Physiol.* 114 1175–1181. 10.1007/s00421-014-2841-x 24563091

[B32] Pontier J. M. Vallée N. Bourdon L. (2009). Bubble-induced platelet aggregation in a rat model of decompression sickness. *J. Appl. Physiol. (Bethesda, Md : 1985)* 107 1825–1829. 10.1152/japplphysiol.91644.2008 19850726

[B33] Pourhashemi S. F. Sahraei H. Meftahi G. H. Hatef B. Gholipour B. (2016). The effect of 20 minutes scuba diving on cognitive function of professional scuba divers. *Asian J. Sports Med.* 7:e38633. 10.5812/asjsm.38633 27826405 PMC5098272

[B34] Santos-Silva A. Rebelo M. I. Castro E. M. Belo L. Guerra A. Rego C. (2001). Leukocyte activation, erythrocyte damage, lipid profile and oxidative stress imposed by high competition physical exercise in adolescents. *Clin. Chim. Acta* 306 119–126. 10.1016/S0009-8981(01)00406-5 11282102

[B35] Skogstad M. Thorsen E. Haldorsen T. Kjuus H. (2002). Lung function over six years among professional divers. *Occup. Environ. Med.* 59 629–633. 10.1136/oem.59.9.629 12205238 PMC1740356

[B36] Smith R. M. Neuman T. S. (1994). Elevation of serum creatine kinase in divers with arterial gas embolization. *N. Engl. J. Med.* 330 19–24. 10.1056/NEJM199401063300104 8259140

[B37] Smith S. M. Heer M. Wang Z. Huntoon C. L. Zwart S. R. (2012). Long-duration space flight and bed rest effects on testosterone and other steroids. *J. Clin. Endocrinol. Metab.* 97 270–278. 10.1210/jc.2011-2233 22049169 PMC3251930

[B38] Stienen G. J. M. (2020). Early adjustments in mitochondrial structure and function in skeletal muscle to high altitude: design and rationale of the first study from the Kilimanjaro Biobank. *Biophys. Rev.* 12 793–798. 10.1007/s12551-020-00710-8 32572680 PMC7429657

[B39] Stowe R. P. Sams C. F. Pierson D. L. (2011). Adrenocortical and immune responses following short- and long-duration spaceflight. *Aviation Space Environ. Med.* 82 627–634. 10.3357/ASEM.2980.2011 21702314

[B40] Sureda A. Batle J. M. Capó X. Martorell M. Córdova A. Tur J. A. (2014). Scuba diving induces nitric oxide synthesis and the expression of inflammatory and regulatory genes of the immune response in neutrophils. *Physiol. Genomics* 46 647–654. 10.1152/physiolgenomics.00028.2014 25005793

[B41] Swenson E. R. Bärtsch P. (2012). High-altitude pulmonary edema. *Compr. Physiol.* 2 2753–2773. 10.1002/cphy.c100029 23720264

[B42] Tetzlaff K. Friege L. Koch A. Heine L. Neubauer B. Struck N. (2001). Effects of ambient cold and depth on lung function in humans after a single scuba dive. *Eur. J. Appl. Physiol.* 85 125–129. 10.1007/s004210100421 11513305

[B43] Verratti V. Bondi D. Jandova T. Camporesi E. Paoli A. Bosco G. (2019). Sex hormones response to physical hyperoxic and hyperbaric stress in male scuba divers: a pilot study. *Adv. Exp. Med. Biol.* 1176 53–62. 10.1007/5584_2019_384 31073929

[B44] Vince R. V. McNaughton L. R. Taylor L. Midgley A. W. Laden G. Madden L. A. (2009). Release of VCAM-1 associated endothelial microparticles following simulated SCUBA dives. *Eur. J. Appl. Physiol.* 105 507–513. 10.1007/s00421-008-0927-z 19002703

[B45] Volozhin A. I. Tsarev V. N. Malneva N. S. Sashkina T. I. Saldusova I. V. (2001). Interaction peculiarities between microbial cenosis and local immunity of periodontium of humans under extreme conditions. *Acta Astronaut.* 49 53–57. 10.1016/S0094-5765(00)00128-4 11858254

[B46] Žarak M. Perović A. Dobrović I. Goreta S. Dumić J. (2020). Galectin-3 and cardiovascular biomarkers reflect adaptation response to scuba diving. *Int. J. Sports Med.* 41 285–291. 10.1055/a-1062-6701 31975358

[B47] Žarak M. Perović A. Njire Bratičević M. Šupraha Goreta S. Dumić J. (2021). Adaptive response triggered by the repeated SCUBA diving is reflected in cardiovascular, muscular, and immune biomarkers. *Physiol. Rep.* 9:e14691. 10.14814/phy2.14691 33463896 PMC7814492

[B48] Zarezadeh R. Azarbayjani M. A. (2014). The effect of air scuba dives up to a depth of 30 metres on serum cortisol in male divers. *Diving Hyperb. Med.* 44 158–160. 25311323

[B49] Zhang K. Wang D. Jiang Z. Ning X. Buzzacott P. Xu W. (2016). Endothelial dysfunction correlates with decompression bubbles in rats. *Sci. Rep.* 6:33390. 10.1038/srep33390 PMC5018851 27615160

[B50] Zhang R. Jia G. Bao J. Zhang Y. Bai Y. Lin L. (2008). Increased vascular cell adhesion molecule-1 was associated with impaired endothelium-dependent relaxation of cerebral and carotid arteries in simulated microgravity rats. *J. Physiol. Sci.* 58 67–73. 18221587 10.2170/physiolsci.RP010707

